# Population Genetic Assessment Model Reveals Conservation Priorities for *Gymnocypris* Species Resources on the Qinghai-Tibetan Plateau

**DOI:** 10.3390/biology13040259

**Published:** 2024-04-14

**Authors:** Jinqiang Quan, Yuling Qu, Yongqing Li, Yue Ren, Guiyan Zhao, Lanlan Li, Junhao Lu

**Affiliations:** 1 College of Animal Science & Technology, Gansu Agricultural University, Lanzhou 730070, China; zhaogy@st.gsau.edu.cn (G.Z.); lujh@st.gsau.edu.cn (J.L.); 2 College of Food Science and Engineering, Gansu Agricultural University, Lanzhou 730070, China; quyul@gsau.edu.cn; 3 Animal Husbandry Quality Standards Institute, Xinjiang Academy of Animal Science, Wulumuqi 830063, China; liyongqing@webmail.hzau.edu.cn; 4 Institute of Livestock Research, Tibet Academy of Agricultural and Animal Husbandry Sciences, Lhasa 850000, China; renyue34@126.com; 5 College of Animal Science & Technology, Ningxia University, Yinchuan 750021, China; llanlan@nxu.edu.cn

**Keywords:** *Gymnocypris* species, genetic diversity, phylogeny, genetic contribution, assessment model, conservation priority

## Abstract

**Simple Summary:**

In this study, we devised two sophisticated models to optimize the conservation assessment of Gymnocypris species germplasm resources, leveraging the genetic diversity and phylogenetic relationships among 674 individuals across eight distinct Gymnocypris species. From the standpoint of genetic diversity, the GPO, GE, and GPR populations have emerged as critical priorities for conservation efforts. This prioritization remains consistent when evaluated from the angle of genetic contribution. Conversely, the perspective of phylogenetic relationships yields a divergent set of priorities for conservation. Ultimately, this research synthesizes the conservation priorities for Gymnocypris genetic resources from multiple analytical perspectives, offering a scientifically grounded framework for formulating conservation strategies specific to Gymnocypris species. This approach is of paramount importance for facilitating the recovery and sustainability of Gymnocypris species resources.

**Abstract:**

The Qinghai-Tibetan Plateau (QTP) has nurtured a rich diversity of species because of its unique geographical and environmental conditions. *Gymnocypris* species (subfamily Schizopygopsinae) are primitive fishes that live in the special environment of the plateau, and their evolution and distribution are inseparable from the historical changes of the QTP. Recently, the resources of *Gymnocypris* species have been decreasing due to habit deterioration and the intensification of human activities. Therefore, the scientific conservation of the genetic resources of *Gymnocypris* species is urgently required. In this study, we established two models for the priority conservation assessment of germplasm resources of *Gymnocypris* species on the basis of the genetic diversity and phylogenetic relationships of 674 individuals from eight *Gymnocypris* species populations. The results show that the *Gymnocypris potanini* (GPO), *Gymnocypris eckloni* (GE), and *Gymnocypris przewalskii* (GPR) populations are the most genetically diverse in terms of combined genetic diversity values and should be prioritized for conservation. In terms of genetic contribution, the GPO, GE, and GPR populations have a positive impact on maintaining the distinctiveness and diversity of the entire *Gymnocypris* species population and should be prioritized for conservation. However, in terms of different evolutionary clades, the *Gymnocypris namensis*, *Gymnocypris waddellii*, *Gymnocypris dobula*, and GE populations in clade A should be given priority for protection, the GE population in clade B should be given priority, and the GPR population in clade C should be given priority. In conclusion, the two models and assessment of conservation priorities will provide a scientific basis for the conservation of *Gymnocypris* species.

## 1. Introduction

The Qinghai-Tibet Plateau (QTP) is the highest plateau in the world and is known as the “Third Pole”. It has unique environmental characteristics because of its special geographical conditions such as high altitude, low temperatures, low oxygen, strong ultraviolet radiation, and large temperature differences between day and night [1]. The QTP is also known as the “Water Tower of the World”, mainly because it has many lakes, glaciers, groundwater, and other water resources, and is the birthplace of many rivers such as the Yangtze, Yellow, Lancang, and Yarlung Tsangpo [2,3]. The exceptional environment and abundant water systems have created a diversity of species, and organisms that inhabit the QTP are well-adapted to the extreme environment [4,5]. Therefore, the genetic information code of these species not only hides the history of geological changes on the QTP, but also preserves traces of the evolutionary expansion and distribution of populations. 

*Gymnocypris* species (subfamily Schizopygopsinae) are native fishes endemic to the QTP. For millions of years, *Gymnocypris* species have evolved to adapt to environmental and geographical changes on the plateau, making them an ideal model for studying the relationship between plateau uplift and adaptation and biological evolution [6,7]. For instance, Zhao et al. (2009) found that the youngest split in sympatric schizothoracine fishes (Cyprinidae) has been shaped by ecological adaptations in a Tibetan Plateau glacier lake through mitochondrial control region sequences and cytochrome b gene analysis [8]. The genetics of *Gymnocypris* fishes on the QTP have been evaluated, and the population diversity of *Gymnocypris* species is correlated with the uplift of the QTP [6]. 

*Gymnocypris* fishes have been able to survive in extremely harsh environments for millions of years and have long been an important and valuable resource for biodiversity [9]. However, with the deterioration in environmental conditions and the expansion of human activities, the resources of *Gymnocypris* species have become endangered, and scientific and rational conservation are urgently required [10]. In this study, we analyzed the genetic diversity of eight *Gymnocypris* species populations on the QTP on the basis of the mitochondrial D-loop region and developed an evaluation model for priority conservation on the basis of genetic diversity, in order to provide a scientifically valid method for the assessment and conservation of *Gymnocypris* species on the QTP.

## 2. Material and Methods

### 2.1. Animal and Sample Collection

In this study, we collected a total of 674 mtDNA D-loop sequences of *Gymnocypris* species from the Tibet and Qinghai, Gansu, and Sichuan Provinces: *Gymnocypris chui*, Tchang, Yueh & Hwang, 1964 (GC), *Gymnocypris scleracanthus* Tsao, C. Z. Wu, Chen & Zhu, 1992 (GS), *Gymnocypris waddellii* Regan, 1905 (GW), *Gymnocypris namensis* Y. F. Wu & M. L. Ren, 1982 (GN), *Gymnocypris dobula* Günther, 1868 (GD), *Gymnocypris przewalskii* Kessler, 1876 (GPR), *Gymnocypris eckloni* Herzenstein, 1891 (GE), and *Gymnocypris potanini* Herzenstein, 1891 (GPO), of which 104 were collected and tested by our laboratory and the other 570 sequences were obtained from the NCBI database. For more information on the sequences, please refer to Supplementary File S1.

### 2.2. DNA Extraction, Amplification, and Sequencing

Genomic DNA was extracted from the caudal fin of *Gymnocypris* species by using the standard phenol/chloroform method [11]. Primers were designed according to the *Gymnocypris przewalskii* partial sequence (AY850398.1) in GenBank (F: 5′ GGG ATA TGT CAT CCT TTA TGG 3′ and R: 5′ GGG TTT GAC AAG AAT AAC AGG 3′). The 20 μL PCR mixture contained 1.0 µL of DNA, 10 µL of 2× GC buffer II, 1 µL of Taq polymerase (2.5 U/µL; Dalian TaKaRa Biotech Co. Ltd., Dalian, China), 1 μL of 10 pmol/mL forward and reverse primers, 2.0 µL of dNTPs (2.5 mM), and 4.0 μL of double-distilled H_2_O (ddH_2_O). The cycling conditions were as follows: initial denaturation at 95 °C for 4 min; 30 cycles of 95 °C for 30 s, 57 °C for 30 s, and 72 °C for 30 s; and a final extension at 72 °C for 10 min. Amplified DNA fragments were purified with agarose gel electrophoresis and sequenced using an ABI 3130 DNA sequencer (Applied Biosystems, Foster City, CA, USA).

### 2.3. Data Analysis

DNA from 104 *Gymnocypris* species individual was amplified using PCR to obtain the 656 bp mtDNA D-loop sequence. The original sequence data obtained by sequencing were edited using Chromas version 2.33 (http://www.technelysium.com.au/chromas.html (accessed on 20 June 2022)), and the correspondence between the electrophoresis peak map and bases was verified. The sequences were aligned using MEGA 7.0 software (https://www.megasoftware.net/ (accessed on 17 July 2022)).

Haplotype diversity (Hd), nucleotide diversity (Pi), average nucleotide variation (K), genetic distance, genetic differentiation coefficient (*Fst*), and gene flow (*Nm*) of the *Gymnocypris* species were calculated using the DnaSP 5.0 software (http://www.ub.edu/dnasp (accessed on 8 August 2022)). A phylogenetic tree was constructed using the maximum likelihood method in MEGA 7.0; the number of bootstrap values was 1000, and the model/method was Kimura 2-parameter. A haplotype median-joining network was constructed with Network 5.0 (http://www.fluxus-engineering.com/sharenet.htm (accessed on 15 October 2022)) to evaluate the haplotype relationships. The Pearson correlations of Hd, Pi, and K were calculated using SPSS 19.0, which was also used for principal component analysis (PCA). 

The ratio of the population genetic contribution was calculated according to the method of Petit et al. [12] and Quan et al. [13]. The method calculates the number of haplotypes for each population, the contribution of each population’s haplotype to genetic variation (R_S(k)_), the contribution of genetic uniqueness (R_D(k)_), and the overall genetic contribution (R_T(k)_), which is a combination of the first two as well as the effect of each population in maintaining population genetic variation (C_RS(k)_%), in maintaining interpopulation genetic differentiation (C_RD(k)_%), and in maintaining overall haplotype richness (C_RT(k)_ %) for the entire *Gymnocypris* species, and the effect of each population in maintaining population genetic variation (C_RS(k)_%), in maintaining interpopulation genetic differentiation (C_RD(k)_%), and overall haplotype richness (C_RT(k)_%) as a combined effect of the first two.

## 3. Results

### 3.1. Genetic Diversity Analysis

A total of 656 bp were analyzed to detect single nucleotide polymorphisms (SNPs). No insertion/deletions (indels) were detected in our 104 novel sequences and 570 downloaded sequences. We identified a total of 357 polymorphic sites (Nps) including 156 singleton variable sites (Svs) and 201 parsimony-informative sites (Pis). Therefore, 240 haplotypes (H) were identified in the eight *Gymnocypris* species populations; Hd was the highest for the GE population (0.986) and lowest for the GS population (0.019). Pi and K were the highest for the GPO population (0.02333 and 15.305, respectively) and lowest for the GS population (0.00003 and 0.019, respectively) (Table 1 and Supplementary File S1). 

The intra-species genetic distances were relatively short for each population and zero for the GS population; with respect to the inter-species genetic distances, the GPR, GE, and GPO populations were genetically distant from the other populations (Table 2). Interestingly, we found that the genetic distance between the GS and GC populations (0.002) was lower than the intraspecific genetic distance (0.003) for the GC population. The results of the *Fst* analysis indicated a moderate degree (*Fst* < 0.15) of genetic differentiation between the GS and GC populations and a high degree (*Fst* > 0.25) of genetic differentiation between the other populations (Table 3). The results of the Nm analysis showed that the gene flow between all populations was relatively low (*Nm* < 1), especially between the GS and GC populations (0.0172; Table 3).

### 3.2. Distribution, Expansion, and Phylogenetic Relationships of Populations

**Figure 1:**
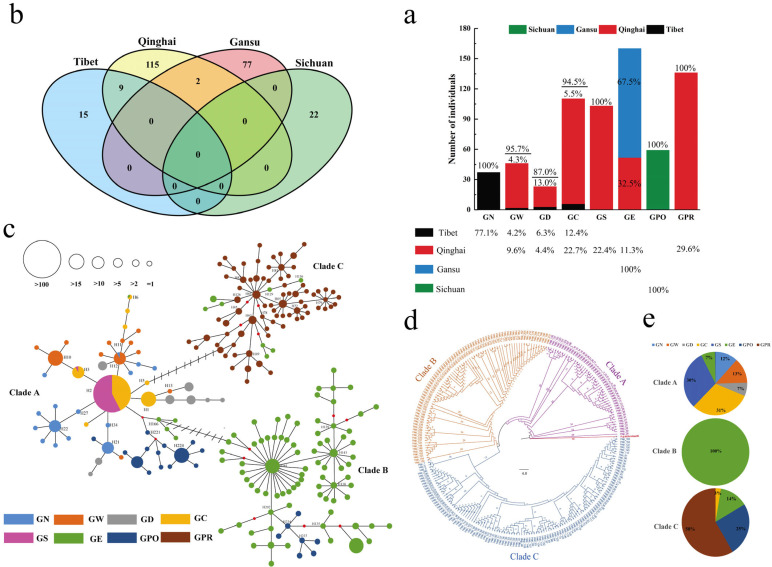
(**a**) Geographical distribution of different *Gymnocypris* species populations on the QTP. (**b**) Uniqueness and shared haplotypes of *Gymnocypris* species in different regions. (**c**) Haplotype network of different *Gymnocypris* species populations. (**d**) Phylogenetic tree for different *Gymnocypris* species populations. (**e**) Distribution of *Gymnocypris* species populations in different evolutionary clades.

The regional distribution of *Gymnocypris* species was measured according to the sample location of each population (Figure 1a). The results showed four *Gymnocypris* species in Tibet, with GN as the dominant population. Six populations are distributed in Qinghai, and the dominant populations are GPR, followed by GC and GS. Gansu and Sichuan have only one population each: GE and GPO, respectively. From a species point of view, the GN population is only found in Tibet; GS and GPR populations only in Qinghai; and the GPO population only in Sichuan; all of them are unique local species. The major parts of the GW, GD, and GC populations are distributed in Qinghai, with a few in Tibet; 67.5% of the GE population is distributed in Qinghai and 32.5% in Gansu.

The analysis of shared and unique haplotypes showed that the largest distribution of unique haplotypes is in the *Gymnocypris* species of Qinghai (115), followed by Gansu (77), Sichuan (22), and Tibet (15). In addition, nine shared haplotypes exist between *Gymnocypris* species of Qinghai and Tibet and two haplotypes with *Gymnocypris* species of Qinghai and Gansu, with no shared haplotypes between the other groups (Figure 1b).

A network of *Gymnocypris* species on the QTP was constructed on the basis of the dominant haplotypes (Figure 1c). The results show that the *Gymnocypris* species form three main clades on the QTP. In clade A, the GC and GS populations are dominant; the GC population showed marked signs of expansion, whereas GS hardly underwent any expansion. In clade B, GE is the dominant population with a significant expansion. In clade C, GPR is the dominant population and has also experienced expansion.

The phylogenetic tree of the *Gymnocypris* species was constructed on the basis of 240 haplotypes, with the Russian sturgeon (*Acipenser gueldenstaedtii*, GenBank: AF238764.1) as the outgroup. The results show that the *Gymnocypris* species have three clades (Figure 1d), which is consistent with the network results. Seven populations are present in clade A, with the GC and GS populations being overwhelmingly dominant, followed by the GW and GN populations (Figure 1e). Four populations exist in clade C, with the GPR population being overwhelming dominant and followed by the GPO, GE, and GC populations. Only the GE population is present in clade B.

### 3.3. Establishment of a Comprehensive Genetic Diversity Evaluation Model

**Figure 2:**
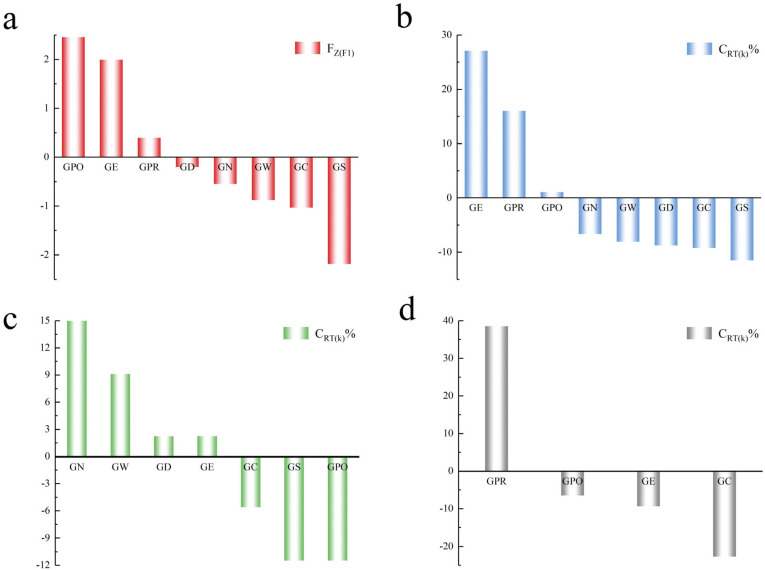
(**a**) Order of priority protection on the basis of combined genetic diversity values (Fz_(F1)_). (**b**) Order of priority protection on the basis of overall genetic contribution (C_RT(k)_ %). (**c**) Order of priority protection on the basis of genetic contribution (C_RT(k)_ %) of clade A. (**d**) Order of priority protection on the basis of genetic contribution (C_RT(k)_ %) of clade C.

To assess the genetic diversity of species scientifically and rationally, we used PCA to establish a model for evaluating the integrated value of genetic diversity. The results showed a significant positive correlation among Hd, Pi, and K (Table 4), indicating that these three parameters can determine the level of population genetic diversity. The main dimensions of Hd, Pi, and K were analyzed using a dimensionality reduction method to extract the principal component values (F1); the cumulative eigenvalues amounted to 81.542%. Hd, Pi, and K were analyzed separately, and their trends were consistent with Fz_(F1)_. Therefore, PCA value Fz_(F1)_ provided sufficient confidence. The results showed that 37.5% of the population presented a positive Fz_(F1)_ of genetic diversity (Figure 2a), among which the highest value was observed for GPO (2.455) and the lowest for GS (−2.188).

### 3.4. Establishment of a Genetic Contribution Rate Assessment Model

Genetic contribution rates were calculated for the eight populations on the basis of the genetic contribution rate model of Quan et al. [13]. The results showed that the GE, GPR, and GPO populations contributed positively to the overall population, whereas the other five populations contributed negatively to the overall population (Table 5). Therefore, the GE, GPR, and GPO populations should be prioritized for conservation (Figure 2b).

Similarly, the genetic contribution of populations in each clade was calculated on the basis of the different evolutionary clades. The results show that in clade A, the GN, GW, GD, and GE populations contributed positively to all populations of clade A (Table 6), whereas the remaining four populations contributed negatively (no GPR population in clade A); therefore, the GN, GW, GD, and GE populations should be protected on a priority basis (Figure 2c). In clade C, the GPR population contributed positively (Table 6), whereas the GC, GE, and GPO populations contributed negatively (no GN, GW, GD, and GS populations in clade C); therefore, the GPR population should be protected on a priority basis (Figure 2d). Clade B only has the GE population, so the GE population should be protected on a priority basis.

## 4. Discussion

### 4.1. Assessment of Gymnocypris Species for Priority Conservation on the Basis of Genetic Diversity

The *Gymnocypris* species belong to the subfamily Schizothoracinae, are endemic to the QTP river system, are relatively slow-growing, tolerant to high salinity and low temperatures, and have reproductive migratory characteristics rarely found in plateau fishes [14]. The distribution and evolution of fishes are limited by water systems, making *Gymnocypris* species an ideal model for biogeographic studies. Recently, the wild resources of *Gymnocypris* species have been declining due to environmental degradation, artificial fishing, and the low recovery capacity of the population itself, so they were listed as endangered on the Red List of Chinese Vertebrates in 2016 [15]. A previous study has shown that *Gymnocypris* species are rich in genetic diversity in terms of morphological characteristics, proteins, genome, and mtDNA, reflecting the exceptional evolutionary history of these plateau fishes and their ability to adapt to the complex environment of the plateau [1]. To scientifically and effectively conserve the germplasm resources of *Gymnocypris* species, it is particularly important to investigate the genetic diversity, population structure, and priority conservation of *Gymnocypris* fishes distributed on the QTP.

In this study, the eight populations showed differences in Hd; the GE population had the highest value, followed by the GPR and GPO populations. The GPO population showed the highest Pi and K values, followed by the GE population. In contrast, the GS population showed the lowest Hd, Pi, and K values. Hd, Pi, and K are three representative parameters for genetic diversity assessment, of which Hd is a measure of the uniqueness of a specific haplotype in a population and thus reflects the abundance of haplotypes in a population. Pi and K reflect only the degree of variation in each haplotype within a population [16]. It is challenging to prioritize conservation on the basis of three separate perspectives. The comprehensive values of genetic diversity that we established using PCA take Hd, Pi, and K into account to judge the level of genetic diversity of different species in an integrated manner. The GPO, GE, and GPR populations had positive Fz_(F1)_ values, indicating that they have relatively high genetic diversity and, conversely, that the other populations have low genetic diversity (Figure 2a).

The GPO population is more widely distributed than the other populations in China such as in the Jinsha and Lancang River systems and in Songpan County in the eastern part of the QTP. Therefore, the GPO population is subject to greater disturbance by artificial selection, which may account for its higher genetic diversity; this is consistent with the findings of similar studies [17]. The GPR population is widely distributed in Qinghai Lake; the GE population is present not only in the Qinghai Lake system but also in the Yellow River system and therefore similarly subject to human intervention, thus allowing these populations to acquire higher genetic diversity [18]. We found that all three populations (GPO, GE, and GPR) had relatively high H, Nps, Pis, and Svs, which are important intrinsic factors that contribute to high genetic diversity. The GS population is distributed in only the Langtze region of Tibet; the population size is small with little interaction with the outside world [19], resulting in its low genetic diversity. According to our investigation, the other populations (GC, GW, GN, and GD) are also geographically homogeneous and isolated and have low H, Nps, Pis, and Svs, resulting in low genetic diversity.

The main indicators of the degree of polymorphism in a population are the genetic distance between populations and the population differentiation index, with higher values of genetic distance and population differentiation index representing higher polymorphism in the population [20]. GPO, GE, and GPR are more genetically distant from the other populations and more genetically differentiated, resulting in a higher haplotype diversity. Interestingly, we found that the genetic distances between the GS and GC populations were lower than those within the GC population. This also indicates a stable GS population structure with very little genetic variation within the species [21]. Therefore, we infer that there is a large genetic divergence between all populations, except for GC and GS, and the presence of genetic divergence between *Gymnocypris* populations makes the recovery of their resources possible. Zhao et al. (2006) studied the structure and genetic diversity of *Gymnocypris* species population in Qinghai Lake on the basis of the mitochondrial *Cyt b* gene, detected frequent gene exchange and low genetic diversity within the population, and speculated that it may have historically experienced a bottleneck effect [22]. Similarly, analysis of the mitochondrial D-loop gene revealed that genetic variation in the Qinghai Lake *Gymnocypris* species was mainly within populations, with less variations between populations; this suggests that the *Gymnocypris* species established a relatively stable pattern of reproductive migration, with greater gene exchange between populations migrating to the same river for mating and less gene exchange between populations migrating to different rivers for breeding [23].

### 4.2. Model for Assessing Genetic Contribution on the Basis of Phylogeny and Genetic Diversity

The phylogeny of a species is in part a response to its genetic variation and is part of the study of genetic diversity. Of the four regions sampled in this study, Qinghai Province has the richest distribution of *Gymnocypris* species resources (both in terms of species and numbers), largely because of the wide distribution of Qinghai Lake and its water system [24]. The trend in network distribution showed that domestication events of *Gymnocypris* species from clades A and C were mainly located in Qinghai, with a trend for clade A to expand from the Qinghai region to the Tibetan region (Figure 1a,c). Clade B had two domestication events distributed in the Qinghai and Gansu regions, mainly in the GE population, and the phylogenetic tree analysis also supports the validity of this view. The GE population was found in the upper reaches of the Yellow River; as the Qinghai Lake and Yellow River were connected in the past, previous studies have suggested that the GE and GPR populations are closely related. However, similar studies have concluded that they do not form a separate lineage, that there is a crossover between their individuals, and the GPR population originated from the GE population [18]. Therefore, it is essential to consider the priority conservation of *Gymnocypris* species from different evolutionary clades.

For the genetic diversity calculations, the frequency of occurrence of each locus and haplotype was averaged (i.e., their contribution to genetic diversity was treated equally) and the contribution of certain specific haplotypes determined by some of these specific loci to both the maintenance and enhancement of population diversity was not revealed. Therefore, to integrate the contributions of both genetic variation and genetic distinctiveness within populations, the method proposed by Petite et al. (1998) and optimized and improved by Quan et al. (2020) to assess the conservation priority of species with a genetic contribution model was adopted. Positive values indicate a positive effect, which means that the genetic contribution of the taxon is higher than the average genetic contribution of the populations, and the presence of the population increases the intra-population genetic variation, inter-population genetic differentiation, or overall population allelic richness. Negative values indicate a negative effect, which means that the genetic contribution of the population is lower than the average genetic contribution of the populations, and the presence of the taxon decreases the intra-population genetic variation, inter-population genetic differentiation, or overall population allelic richness [13].

Thus, the GE, GPO, and GPR populations have a positive impact on the entire population of *Gymnocypris* species in terms of genetic contribution, indicating that they have a positive role in maintaining population diversity; therefore, these populations should be prioritized for conservation. In contrast, other populations play a negative role in maintaining the genetic diversity of the entire population (Figure 2b). On the basis of the different clades of the phylogenetic tree, the GN, GW, GD, and GE populations contribute positively to the genetic diversity of all populations on clade A; therefore, they should be prioritized for conservation. The GE population is unique in clade B, so it should also be protected on a priority basis. The GPR population plays a positive role in the diversity of the other three populations in clade C, so it should be prioritized for protection. Therefore, without considering the systematic variability of alleles, we suggest that the GE and GPR populations be grouped into the same conservation unit because they are in the same geographic pattern and political region or distribution, and that the GPOs be grouped into a separate conservation unit [25].

## 5. Conclusions

In this study, two models were developed for the priority conservation evaluation of *Gymnocypris* species on the basis of their genetic diversity and genetic contribution. The order of conservation priorities for *Gymnocypris* populations in terms of genetic resources was considered from different perspectives, which will provide a reference for the development of conservation strategies for *Gymnocypris species* and is of great scientific significance for the restoration of *Gymnocypris* species resources.

## Figures and Tables

**Table 1:** Genetic diversity of *Gymnocypris* species on the QTP.

Abbreviation	S	H	Nps	Pis	Svs	Hd	Pi	K
GC	110	8	20	19	1	0.500 ± 0.053	0.00307	2.013
GS	103	2	1	0	1	0.019 ± 0.019	0.00003	0.019
GW	44	12	12	6	6	0.718 ± 0.058	0.00187	1.227
GN	39	15	15	6	9	0.833 ± 0.043	0.00295	1.938
GD	23	8	9	8	1	0.858 ± 0.044	0.00511	3.352
GPR	136	77	86	35	51	0.985 ± 0.003	0.00795	5.207
GE	159	100	140	65	75	0.986 ± 0.004	0.01907	12.452
GPO	60	24	74	62	12	0.880 ± 0.030	0.02333	15.305

Note: S: size of population; H: number of haplotypes; Nps: number of polymorphic sites; Pis: parsimony informative sites; Svs: singleton variable sites; Hd: haplotype diversity; Pi: nucleotide diversity; K: average number of nucleotide differences.

**Table 2:** Genetic distances of *Gymnocypris* species on the QTP.

Population	GC	GS	GW	GN	GD	GPR	GE	GPO
GC	0.003							
GS	0.002	0						
GW	0.004	0.003	0.002					
GN	0.005	0.003	0.003	0.003				
GD	0.007	0.006	0.005	0.006	0.005			
GPR	0.033	0.034	0.031	0.033	0.031	0.008		
GE	0.024	0.024	0.024	0.025	0.025	0.028	0.02	
GPO	0.031	0.031	0.031	0.033	0.034	0.04	0.036	0.023

Note: Diagonal lines are the intra-population genetic distances. Lower left is the inter-population genetic distance.

**Table 3:** Genetic differentiation coefficient and gene flow of *Gymnocypris* species on the QTP.

Population	GC	GS	GW	GN	GD	GPR	GE	GPO
GC		0.0172	0.0598	0.0585	0.0596	0.0346	0.0624	0.0612
GS	0.0743		0.0553	0.0618	0.0619	0.0261	0.0610	0.0589
GW	0.3961	0.6695		0.0494	0.0574	0.0328	0.0620	0.0595
GN	0.3738	0.5529	0.2713		0.0547	0.0342	0.0620	0.0595
GD	0.3917	0.5504	0.3569	0.3239		0.0415	0.0625	0.0607
GPR	0.8338	0.8817	0.8446	0.8366	0.7901		0.0625	0.0596
GE	0.5153	0.5775	0.5439	0.5469	0.5024	0.4982		0.0602
GPO	0.5718	0.6198	0.5990	0.6088	0.5845	0.6071	0.4044	

Note: The genetic differentiation coefficient (*Fst*) is shown in the lower left corner and gene flow (*Nm*) in the upper right corner.

**Table 4:** Correlation matrix between the three genetic diversity indices.

Item	Hd	Pi	K
Hd	1.000		
Pi	0.568 **	1.000	
K	0.568 **	1.000 **	1.000

Note: ** Significantly correlated at the 0.01 level (both sides); Hd: haplotype diversity; Pi: nucleotide diversity; K: average number of nucleotide differences.

**Table 5:** Overall genetic contribution of *Gymnocypris* species on the QTP.

Population	R_S(k)_	R_D(k)_	R_T(k)_	C_RS(k)_%	C_RD(k)_ %	C_RT(k)_ %
GN	1.000	12.625	13.625	−1.1560	−5.5132	−6.6692
GW	0.250	10.000	10.250	−1.4609	−6.5803	−8.0412
GD	1.500	7.000	8.500	−0.9527	−7.7998	−8.7525
GC	1.875	5.500	7.375	−0.8003	−8.4096	−9.2099
GS	1.000	0.750	1.750	−1.1560	−10.3404	−11.4964
GE	9.625	87.000	96.625	2.3501	24.7205	27.0706
GPO	12.500	20.125	32.625	3.5188	−2.4644	1.0544
GPR	3.000	66.500	69.500	−0.3430	16.3872	16.0442

Notes: R_S(k)_, contribution of genetic diversity; R_D(k)_, contribution of genetic distinctiveness; R_T(k)_, contribution of total genetics of the *k*th population; C_RS(k)_%, rates of contribution attributed to genetic variation; C_RD(k)_ %, rates of contribution attributed to genetic distinctiveness; C_RT(k)_ %, rates of total genetic contribution rate of the *k*th population.

**Table 6:** Genetic contributions of different clades of *Gymnocypris* species on the QTP.

Population	Clade A						Clade C					
	R_S(k)_	R_D(k)_	R_T(k)_	C_RS(k)_%	C_RD(k)_ %	C_RT(k)_ %	R_S(k)_	R_D(k)_	R_T(k)_	C_RS(k)_%	C_RD(k)_ %	C_RT(k)_ %
GN	2.143	12.357	14.500	2.1609	12.8252	14.9861						
GW	1.714	9.786	11.500	1.3206	7.7832	9.1037						
GD	1.143	6.857	8.000	0.2001	2.0409	2.2410						
GC	0.714	3.286	4.000	−0.6402	−4.9619	−5.6022	0.750	1.750	2.500	−5.6250	−17.0833	−22.7083
GS	0.286	0.714	1.000	−1.4806	−10.0039	−11.4845						
GE	1.143	6.857	8.000	0.2001	2.0409	2.2410	4.750	13.750	18.500	−2.2917	−7.0833	−9.3750
GPO	0.143	0.857	1.000	−1.7607	−9.7238	−11.4845	5.500	16.500	22.000	−1.6667	−4.7917	−6.4583
GPR							19.000	57.000	76.000	9.5833	28.9583	38.5417

## Data Availability

Sequences of the mtDNA D-loop of 104 individuals of *Gymnocypris* species were submitted to GenBank (Accession numbers KJ610703-KJ610806).

## Supplementary Materials

The following supporting information can be downloaded at: https://www.mdpi.com/article/10.3390/biology13040259/s1, File S1: Sequence information for mtDNA D-loop of 674 individuals of *Gymnocypris* species.
